# Evaluating Perfusion Quantification for Phase‐Resolved Functional Lung MRI Using Gaussian Filtering

**DOI:** 10.1002/nbm.70420

**Published:** 2026-10-13

**Authors:** Kirsten J. Schmidt, Andreas Voskrebenzev, Julian M. W. Glandorf, Marius M. Klein, Lea Behrendt, Frank Wacker, Jens Vogel‐Claussen, Filip Klimeš

**Affiliations:** ^1^ Institute of Diagnostic and Interventional Radiology Hannover Medical School Hannover Germany; ^2^ Biomedical Research in Endstage and Obstructive Lung Disease Hannover (BREATH), Member of the German Centre for Lung Research Hannover Germany; ^3^ Department of Radiology Charité‐Universitätsmedizin Berlin, Corporate Member of Freie Universität Berlin and Humboldt‐Universität zu Berlin Berlin Germany; ^4^ Department of Radiation Protection and Medical Physics Hannover Medical School Hannover Germany

**Keywords:** DCE‐MRI, perfusion, PREFUL MRI, pulmonary MRI, repeatability, validation

## Abstract

Phase‐resolved functional lung (PREFUL) MRI enables contrast‐free lung perfusion quantification (Q_Quant_). Current quantification uses a single median value per slice of maximal blood exchange fraction (ΔS), which limits regional assessment, whereas regional ΔS causes artifacts. This retrospective study aims to optimize Q_Quant_ by applying Gaussian filtering to ΔS to improve intrascan/interscan repeatability in healthy volunteers, while evaluating whether the optimized Q_Quant_ retains agreement with dynamic contrast‐enhanced (DCE) MRI in chronic obstructive pulmonary disease (COPD) and chronic thromboembolic pulmonary hypertension (CTEPH) patients and exhibits a physiologically plausible gravity‐dependent perfusion gradient. Analysis was performed on 29 healthy subjects (11 female, age: 19–33 years), 20 COPD patients (three female, age: 46–76 years) and 15 CTEPH patients (six female, age: 44–82 years), scanned at 1.5 T. Healthy subjects underwent four PREFUL scans in two sessions. Patients just underwent one PREFUL and DCE scan. Q_Quant_ maps were calculated while applying different Gaussian filter options to ΔS. In patients, PREFUL‐derived Q_Quant_ maps were coregistered to DCE‐derived pulmonary blood flow (PBF) maps for global and voxel‐wise correlations. In healthy subjects, the gravity‐dependent perfusion gradient was quantified by linear regression of slice‐wise Q_Quant_ across five coronal slices. Gaussian filtering ΔS with *σ* = 1.5 improved intrascan and interscan repeatability in healthy subjects (mean bias: ≤ 2.94 mL/100 mL; Cohen's *d*: 0.2) compared with the current slice median setting (mean bias: ≥ 3.75 mL/100 mL; Cohen's *d*: 0.68). Correlations with PBF remained stable after filtering (σ = 1.5) in COPD (filtered vs. default ΔS: global *r* = 0.57 vs. *r* = 0.59, voxel‐wise *r* = 0.32 vs. *r* = 0.32) and CTEPH (global *r* = 0.32 vs. *r* = 0.28, voxel‐wise *r* = 0.40 vs. *r* = 0.41), all significant except global CTEPH. The filtered Q_Quant_ maps showed a significant gravity‐dependent perfusion gradient (ΔS filtered *σ* = 1.5) of 1.47 mL/min/100 mL/cm. Gaussian filtering ΔS with *σ* = 1.5 improved Q_Quant_ repeatability while preserving DCE‐MRI agreement and is therefore recommended. The observed gravity‐dependent perfusion gradient confirms physiological plausibility, supporting potential in clinical use.

## Graphical abstract

Current PREFUL perfusion quantification uses a slice‐wise median for the maximal blood exchange fraction ΔS, limiting regional information. Gaussian filtering (*σ* = 1.5) ΔS in perfusion calculation adds regional information and improves intrascan and interscan repeatability while preserving global and voxel‐wise correlations with DCE‐derived pulmonary blood flow.

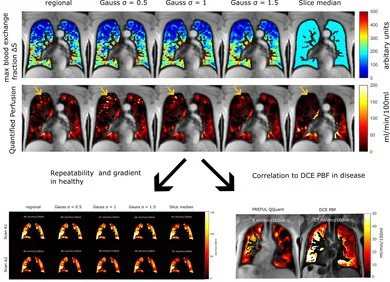

**Abbreviations**
ANTs: advanced normalization tools
BW: bandwidth
COPD: chronic obstructive pulmonary disease
CoV: coefficient of variation
CTEPH: chronic thromboembolic pulmonary hypertension
DCE: dynamic contrast enhanced
FA: flip angle
FEV_1_
: forced expiratory volume in 1 s
FVC: forced vital capacity
GF: Gaussian filter
PBF: pulmonary blood flow
PREFUL: phase‐resolved functional lung
Q_Quant_
: quantified PREFUL perfusion
QQuant_norm_
: quantified PREFUL perfusion normalized for heart frequency
ST: slice thickness
ΔS: maximal blood exchange fraction

## 1 Introduction

Impaired pulmonary blood flow (PBF) is a hallmark of many chronic lung diseases, including the highly prevalent chronic obstructive pulmonary disease (COPD) and the rarer chronic thromboembolic pulmonary hypertension (CTEPH). Current guidelines [1, 2] recommend spirometry and ventilation‐perfusion single‐photon emission computed tomography (V/Q SPECT) measurements for assessment. Spirometry is noninvasive but provides only global lung parameters without spatial information. Established methods such as CT or SPECT can offer spatial resolution, but its utility in follow‐up examinations is limited by cumulative radiation exposure.

MRI enables regional, ionizing radiation‐free evaluation of pulmonary function and has emerged as a promising alternative. Both contrast‐enhanced techniques (e.g., dynamic contrast‐enhanced [DCE] MRI) and contrast‐free approaches (e.g., arterial spin labelling [ASL] and Fourier decomposition‐based methods like phase‐resolved functional lung [PREFUL] MRI) [3] are increasingly being explored [4].

DCE‐MRI is a clinically established method for quantitative pulmonary perfusion assessment [4]. It images the first pass of a gadolinium‐based contrast agent bolus during breath hold in a 3D volume with high temporal resolution and calculations of the signal evolution in T1‐weighted sequences [5, 6]. Despite its clinical utility, DCE‐MRI has limitations. It requires contrast agent administration, which may be contraindicated in certain patients and raises concerns regarding long‐term gadolinium deposition in brain tissue [7]. Additionally, it demands breath hold that certain patients cannot sustain for the entire measurement.

In contrast, PREFUL MRI enables contrast‐free, free‐breathing assessment of pulmonary perfusion by exploiting signal variations during the cardiac cycle. Acquisition uses a conventional spoiled gradient echo sequence. PREFUL‐derived quantification of perfusion (Q_Quant_) relies on proton density and signal decay across multiple excitations towards steady state [8]. Using the signal (S) in parenchyma (par) and vessels (ves) at mid‐respiration position, at time = 0 (S0_par_/S0_ves_) and the median steady‐state parenchyma signal (S1_par_), the exchanged spin fraction per heartbeat and blood proportion per voxel are estimated. Subsequently, Q_Quant_ is calculated according to the following formula [8]:

$$\begin{array}{c} Q_{\text{Quant}}=\text{exchange fraction}\cdot \text{blood fraction}\cdot \\ \;\text{voxel volume}\cdot \frac{1}{T}\cdot \frac{100}{\text{voxel volume}}, \end{array}$$

with the voxel volume cancelling out, # indicating global slice values, *T* the time between two heartbeats, and *Q* the signal amplitude during heart cycle resulting in:

$$Q_{\text{Quant}}=\frac{Q}{(S0_{\mathrm{par}}-S1_{\mathrm{par})\#}}\cdot \frac{S0_{\mathrm{par}}}{S1_{\mathrm{ves}}\#}\cdot \frac{1}{T}\cdot 100,$$

The maximal blood exchange fraction can be shortened to $\mathrm{\Delta S}=S0_{\mathrm{par}}-S1_{\mathrm{par}}$. A more detailed explanation of all parameters can be found in the paper by Glandorf et al. [8].

A limitation of the current approach is that ΔS is estimated as a global slice‐wise median value (ΔS_median_). While this median approach improves robustness against noise, it may obscure regional heterogeneity in diseased lungs. Conversely, voxel‐wise estimation of ΔS can better capture spatial inhomogeneities but is highly susceptible to noise due to low signal‐to‐noise ratio [8].

Beyond these methodological issues, evaluation gaps remain. The repeatability and physiological validity of Q_Quant_ have not been systematically evaluated in healthy subjects. A perfusion gradient using PREFUL has only been assessed in perfusion‐weighted maps with arbitrary units [9].

Therefore, this study aims to (a) optimize ΔS estimation using Gaussian filtering to improve intrascan and interscan repeatability of Q_Quant_ in healthy volunteers; (b) evaluate the agreement of PREFUL‐derived perfusion with clinically established DCE‐MRI measurements in patients with COPD and CTEPH; and (c) assess the ability of Q_Quant_ to capture the physiologically plausible gravity‐dependent perfusion gradient [10], in healthy individuals.

## 2 Materials and Methods

### 2.1 Study Participants

This retrospective analysis was conducted under a protocol approved by the Institutional Review Board. All participants provided informed written consent participating in the study. The participants consisted of three cohorts: a healthy cohort for repeatability, and two disease cohorts, COPD and CTEPH, used for validation with DCE. The healthy repeatability cohort comprised 29 healthy subjects (11 female, age range: 19–33) from a previous study [11]. The validation cohorts comprised 20 patients with COPD (13 female, age range: 46–76), representing a subcohort of the CLAIM Study [12] and 15 patients with CTEPH (six female, age range: 44–82). Patients for the COPD cohort were eligible if they were aged ≥ 40 years, had a confirmed clinical diagnosis of COPD, a smoking history of at least 10 years, airflow limitation (baseline postbronchodilator forced expiratory volume in 1s (FEV_1_) of less than 80% predicted and a postbronchodilator FEV_1_‐to‐forced vital capacity (FVC) ratio of less than 0.7), hyperinflation (residual volume > 135% predicted), and stable cardiovascular function. Arrhythmia, heart failure (left ventricular ejection fraction < 40%), unstable ischaemic heart disease, and uncontrolled hypertension were exclusion criteria. Patients with CTEPH were eligible if they were aged ≥ 18 years and had pulmonary hypertension diagnosed by transthoracic echocardiography.

Exclusion criteria for both groups were incomplete MRI protocols and any contraindications to MRI or gadolinium‐based contrast agents.

To further characterize subjects, spirometry measurements were performed between repeatability scans in the healthy cohort and after the MRI scans of the COPD cohort. Outcomes were FEV_1_, FVC, and Tiffeneau index (FEV_1_/FVC), obtained according to guidelines [13]. In the CTEPH cohort, no spirometry measurements were performed.

### 2.2 Image Acquisition

Imaging of the healthy repeatability cohort was performed at 1.5 T (MAGNETOM Avanto, Siemens Healthineers, Erlangen, Germany). Subjects underwent two PREFUL MRI sessions (A and B), separated by ~1 h, acquiring the same five coronal slices (250 images each) twice (A1/2 and B1/2) with a 2D spoiled gradient echo sequence with the following settings: field of view (FOV): 500 × 500 mm^2^, matrix size: 128 × 128, slice thickness (ST): 15 mm, echo time (TE): 0.82 ms, repetition time (TR): 3 ms, flip angle (FA): 5°, and bandwidth (BW): 1500 Hz/px.

The PREFUL and DCE scans of the COPD/CTEPH validation cohort were performed at 1.5 T (MAGNETOM Avanto or Aera, Siemens Healthineers, Erlangen, Germany). For PREFUL, three coronal slices (200–250 images each, at tracheal bifurcation, anterior and posterior to the tracheal bifurcation) were acquired with the following settings: FOV: 340 × 340 to 500 × 500 mm^2^, matrix size: 128 × 96 or 128 × 128, ST: 15 mm, TE: 0.67–0.91 ms, TR: 3 ms, FA: 5°–6°, and BW: 1085–1502 Hz/px.

These PREFUL scans were directly followed by DCE examinations acquired in a single breath hold at end inspiration, using a bolus of 0.033–0.04‐mmol/kg bodyweight of gadolinium‐based contrast agents injected at a rate of 5 mL/s, using a 3D time‐resolved angiography with stochastic trajectories (TWIST) sequence with the following settings: FOV: 276 × 340 to 421 × 500 mm^2^, matrix size: 192 × 113 to 156 × 146, ST: 3–6 mm, TE: 0.7–0.89 ms, TR: 2.13–2.74 ms, and BW: 570–744 Hz/px. An overview of scan parameters is presented in Table 1.

**TABLE 1:** Sequence parameters of PREFUL MRI and DCE‐MRI.

Parameters	PREFUL scan healthy repeatability	PREFUL scan COPD	PREFUL scan CTEPH	DCE scan COPD/CTEPH
Sequence	Spoiled gradient echo	Spoiled gradient echo	Spoiled gradient echo	TWIST
FOV [mm^2^]	500 × 500	500 × 500	340 × 340–500 × 500	276 × 340–421 × 500
Matrix size	128 × 128	128 × 96	128 × 96–128 × 128	192 × 113–156 × 146
Slice thickness [mm]	15	15	15	3–6
TE [ms]	0.82	0.82	0.82–0.87	0.7–0.89
TR [ms]	3	3	3	2.13–2.74
Flip angle [°]	5	5	5–6	10–30
Pixel bandwidth [Hz/px]	1500	1500	1085–1502	570–744
Images per slice (PREFUL)/temporal resolution (DCE)	250	200	200–250	0.8–1.1 s
Number of slices	5	3	3	

Abbreviations: COPD, chronic obstructive pulmonary disease; CTEPH, chronic thromboembolic pulmonary hypertension; DCE, dynamic contrast‐enhanced MRI; PREFUL, phase‐resolved functional lung; TWIST, time‐resolved angiography with stochastic trajectories.

### 2.3 PREFUL MRI Postprocessing and Image Analysis

All acquired PREFUL images were registered towards a middle respiratory image using advanced normalization tools (ANTs) [14]. Subsequently, registered images were high‐pass (cutoff frequency of 0.75 Hz) filtered to maintain the perfusion information only. Using a piecewise sine‐fit in an automatically selected high perfused region of interest, e.g., aorta, images were sorted into one cardiac cycle. The detailed cardiac cycle reconstruction has been described previously [3, 15]. Lung parenchyma and, in a separate mask, vessels were segmented with convolutional neural networks [16] and subsequently verified and manually corrected if needed. The parameters of the Q_Quant_ formula, hence Q, S0_par_, S0_ves_, S1_par_, and heart frequency, were calculated as described [8]. Five Q_Quant_ maps were then calculated for each subject, each derived from a different version of ΔS (the signal decay in the parenchyma ROI). The first map used the current default approach, a single representative ΔS value per slice within the lung parenchyma (ΔS_median_). For the second map, a regional approach without any filtering was applied (ΔS_regional_). The remaining three maps were obtained by applying Gaussian filtering to ΔS maps with standard deviations of *σ* = 0.5, *σ* = 1.0, and *σ* = 1.5, denoted ΔS_GF0.5_, ΔS_GF1_, and ΔS_GF1.5_, respectively.

### 2.4 DCE‐MRI Postprocessing

Postprocessing of the DCE‐MRI began by identifying and selecting the perfusion weighted phase corresponding to the arrival of the contrast bolus in the aortic arch. The DCE maps were then denoised by applying a 3D Gaussian filter [15, 17]. Subsequently, DCE‐MRI derived PBF maps were calculated using a deconvolution algorithm based on the Volterra formula for discretization, with singular value decomposition applied as regularization [5, 6].

### 2.5 Intrascan and Interscan Repeatability of Q_Quant_ Under Varying ΔS Application in Healthy Volunteers

For the healthy repeatability cohort, all Q_Quant_ maps were normalized for heart frequency (QQuant_norm_), to enable comparison of parameters independent of changing heart frequency (individual heart rates are provided in Table S2). The normalized formula being $Q_{\text{Quant}}=\frac{Q}{(S0_{\mathrm{par}}-S1_{\mathrm{par})\#}}x\frac{S0_{\mathrm{par}}}{S1_{\mathrm{ves}}\#}\;x\;100$. Repeatability was assessed both within a single scan session (intrascan, A1 vs. A2) and between sessions separated by time outside the scanner (interscan, A1 vs. B1).

### 2.6 Comparison of PREFUL and DCE‐MRI

Compound DCE slices to match PREFUL ST were calculated by weighting them according to their overlap with the corresponding PREFUL slices and summed up as described previously [15]. PREFUL images were then registered onto the compound DCE slices using a combination of rigid and nonrigid ANTs transformations. The PREFUL parenchyma mask (excluding large vessels) was registered along to the DCE slice and corrected manually to be used for PREFUL and DCE. The corresponding PREFUL Q_Quant_ maps for all ΔS settings and DCE PBF maps were compared using median global patient values and voxel‐wise correlations. Voxel‐wise correlations were calculated separately for each patient, and the median correlation across all patients was reported.

### 2.7 Perfusion Gradient in Anterior–Posterior Direction

For perfusion gradient calculations, we used only the lung boundary segmentation from the PREFUL pipeline. This segmentation excludes only large pulmonary vessels. The additional PREFUL vessel segmentation was omitted to stay consistent with previous literature, where vessels either were not segmented inside the lung parenchyma [18] or sagittal images with less prone vessels were used [10, 19]. The anterior–posterior perfusion gradient was quantified in the A1 scans of the healthy repeatability cohort. For each healthy volunteer, we performed a linear regression of slice‐wise Q_Quant_ values. The median of all individual slopes was then taken as estimate of the overall gravity‐dependent gradient.

### 2.8 Statistical Analysis

Normality was evaluated by Shapiro–Wilk test and confirmed visually by histogram and Q–Q plot inspection.

As some of the repeatability datasets exhibited deviations from normal distribution, a nonparametric statistical approach was selected to ensure comparability across methods.

In the healthy volunteer's cohort, intrascan and interscan repeatability of Q_Quant_ maps with different ΔS settings was evaluated using coefficient of variation (CoV), intraclass correlation coefficient (ICC), and Cohen's *d*. The differences between the repeated measurements were quantified by Bland–Altman analysis and tested for significance using Wilcoxon signed‐rank test. Alpha = 0.05 was chosen as significance level and Bonferroni adjusted due to the five ΔS methods to alpha = 0.01.

In the COPD and CTEPH cohorts, global and voxel‐wise relationship between DCE‐MRI PBF and PREFUL‐derived Q_Quant_ maps was assessed by Pearson correlation, and the agreement between their median values was quantified using Bland–Altman plots.

Statistical difference between posterior and anterior slice in gradient calculation was assessed by two‐sided paired *t*‐test.

## 3 Results

Demographic and spirometric characteristics of all three cohorts are presented in Table 2.

**TABLE 2:** Overview of study cohorts and their respective median spirometry results.

Cohort	*n* (% female)	Age (years)	FEV_1_% pred.	FVC % pred.	FEV_1_/FVC % pred.
Healthy	29 (37%)	27.1 (5)	100 (15)	98 (14.5)	96 (10.5)
COPD	20 (15%)	65.5 (14)	47 (25)	99 (39)	47 (17)
CTEPH	15 (40%)	56.0 (14)	—	—	—

*Note:* For the CTEPH cohort, no spirometry measurements were performed. Data are presented as *n* (%) or median (IQR).

Abbreviations: COPD, chronic obstructive pulmonary disease; FEV_1_, forced expiratory volume in 1 s; FVC, forced vital capacity.

### 3.1 Intrascan and Interscan Repeatability of Q_Quant_ Under Varying ΔS Conditions in Healthy Volunteers

Figure 1 shows Q_Quant_ maps illustrating intrascan repeatability for each filter option of a middle slice of a representative subject. General visual matching of highly perfused areas was given for all filter options for intrascan repeatability, with baseline A1 median Q_Quant_ ranging from 62 to 68 mL/min/100 mL and intrascan A2 from 54 to 59 mL/min/100 mL. Similarly, to the results of A2, the interscan B1 Q_Quant_ was significantly lower than the baseline, with median Q_Quant_ between 47 mL/min/100 mL (ΔS_median_) and 62 mL/min/100 mL (ΔS_regional_).

**FIGURE 1:**
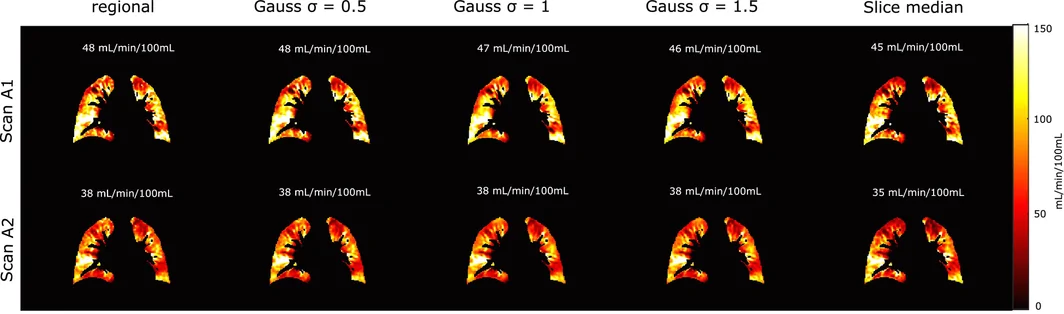
Q_Quant_ maps at baseline (A1) and intrascan repeatability scans (A2) of a 30‐year‐old female healthy volunteer (FEV_1_ = 102% pred.). Global slice median Q_Quant_ values are indicated. Applying a Gaussian filter with *σ* = 1.5 reduced the bias between the repeated measurements.

Among all ΔS options, QQuant_norm_ with ΔS_GF1.5_ provided the highest repeatability, with the lowest mean bias (intrascan: 2.94 mL/100 mL, interscan: 2.93 mL/100 mL) and smallest effect size (Cohen's *d* = 0.20/0.21). In contrast, the currently used ΔS_median_ approach exhibited the lowest repeatability, with the largest effect size (both intrascan and interscan Cohen's *d* = 0.68) and a higher mean bias of 5.21 mL/100 mL (intrascan) or 3.75 mL/100 mL (interscan). The voxel‐wise regional ΔS_regional_ showed small‐to‐moderate effect sizes (*d* = 0.36 intrascan, *d* = 0.24 interscan) and the highest intrascan mean bias (5.65 mL/100 mL) and second highest interscan mean bias (3.34 mL/min). Median CoV remained low across all methods, ranging from 8.33% to 9.62% intrascan and from 10.70% to 12.60% interscan, showing slightly higher variability in interscan measurements. See Table 3 for complete repeatability results. Results without normalization are to be found in Table S3. Bland–Altman analysis is provided in Figure S2.

**TABLE 3:** Median QQuant_norm_, normalized by heart frequency, in intrascan (A) and interscan (B) repeatability scans.

(A) Intrascan							
ΔS Option	Median QQuant_norm_ A1 [mL/100 mL] (IQR)	Median QQuant_norm_ A2 [mL/100 mL] (IQR)	Median CoV [%] (IQR)	Mean bias [mL/100 mL]	Wilcoxon (*p* value)	Cohen's *d*	ICC
Voxel‐wise regional ΔS_regional_	69 (27)	59 (16)	8.33 (10.67)	5.65	0.04	0.36	0.58
GF *σ* = 0.5 ΔS_GF0.5_	67 (26)	58 (15)	9.62 (9.20)	3.28	0.21	0.22	0.61
GF *σ* = 1 ΔS_GF1_	66 (25)	57 (15)	8.93 (10.82)	3.11	0.24	0.21	0.61
GF *σ* = 1.5 ΔS_GF1.5_	65 (25)	57 (15)	8.96 (10.90)	2.94	0.30	0.20	0.61
Slice median ΔS_median_	62 (27)	55 (14)	8.91 (7.22)	5.21	0.06	0.68	0.69
(B) Interscan							
**ΔS Option**	**Median QQuant**_**norm**_ **A1 [mL/100 mL] (IQR)**	**Median QQuant**_**norm**_ **B1 [mL/100 mL] (IQR)**	**Median CoV [%] (IQR)**	**Mean bias [mL/100 mL]**	**Wilcoxon** (***p*** **value**)	**Cohen's** ***d***	**ICC**
Voxel‐wise regional ΔS_regional_	69 (27)	62 (24)	10.75 (11.26)	3.34	0.42	0.24	0.67
GF *σ* = 0.5 ΔS_GF0.5_	67 (26)	58 (24)	12.60 (12.26)	3.01	0.36	0.21	0.64
GF *σ* = 1 ΔS_GF1_	66 (25)	58 (23)	12.24 (12.36)	2.99	0.37	0.21	0.64
GF *σ* = 1.5 ΔS_GF1.5_	65 (25)	57 (23)	11.85 (12.51)	2.93	0.40	0.21	0.65
Slice median ΔS_median_	62 (27)	57 (20)	10.70 (11.46)	3.75	0.29	0.68	0.69

Abbreviations: CoV, coefficient of variation; GF, Gaussian filter; ICC, intraclass correlation coefficient; IQR, interquartile range; QQuant_norm_, quantified perfusion normalized by heart frequency; ΔS, maximal blood exchange fraction.

### 3.2 Comparison of PREFUL and DCE‐MRI

Representative ΔS and Q_Quant_ maps of a CTEPH and a COPD patient are shown in Figure 2. Representative ΔS and Q_Quant_ maps of a healthy volunteer are represented in Figure S1. ΔS_regional_ and filtering with lower kernel sizes, such as ΔS_GF0.5_, resulted in persistent artifacts in Q_Quant_ maps. Visual agreement between DCE PBF and PREFUL Q_Quant_ was generally good in COPD patients (Figure 3, top). However, in CTEPH patients, PREFUL Q_Quant_ frequently underestimated perfusion in large lung regions that appeared perfused on DCE PBF (Figure 3, bottom).

**FIGURE 2:**
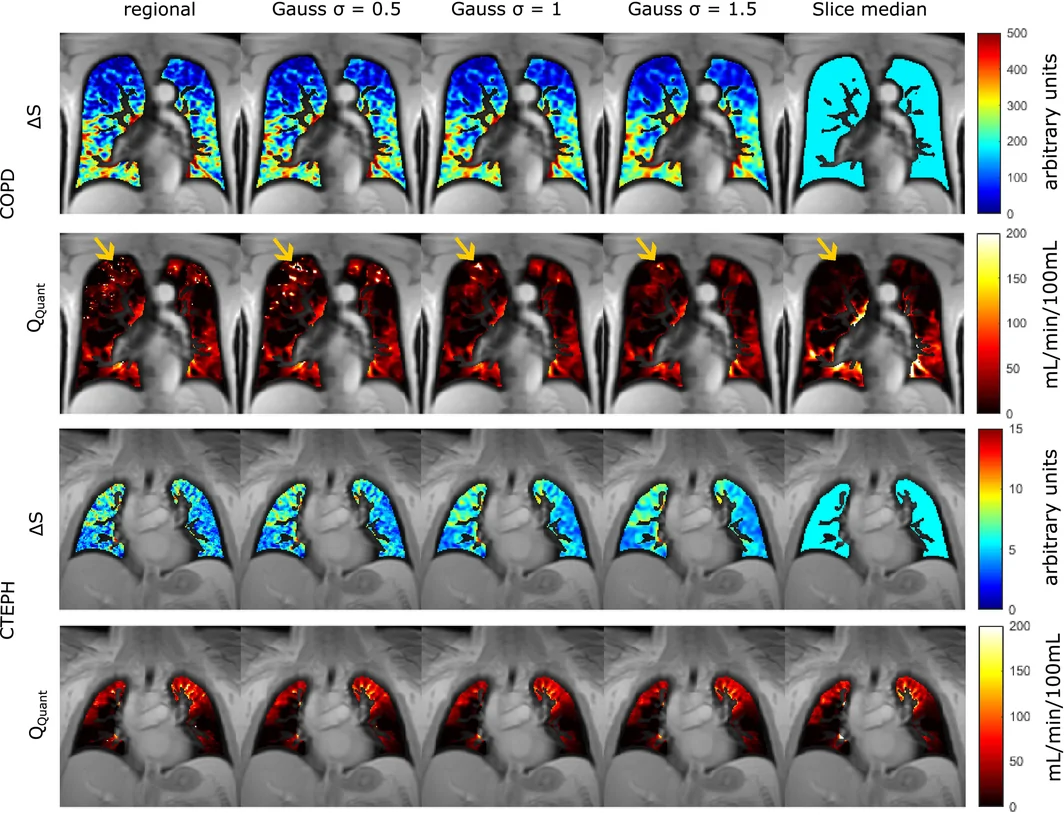
ΔS and Q_Quant_ maps from an exemplary COPD patient (66 years, FEV_1_ = 27% pred.) and a CTEPH patient (59 years) demonstrating the effects of different filter settings during ΔS calculation. Voxel‐wise regional ΔS calculation exhibits artifacts (minimal or no signal decay; examples indicated by yellow arrows) in Q_Quant_ that are most pronounced in COPD and only minor in CTEPH. Increasing the strength of the Gaussian filter progressively reduces these artifacts, and applying either a *σ* = 1.5 Gaussian filter or a slice‐wise median ΔS completely eliminates them.

**FIGURE 3:**
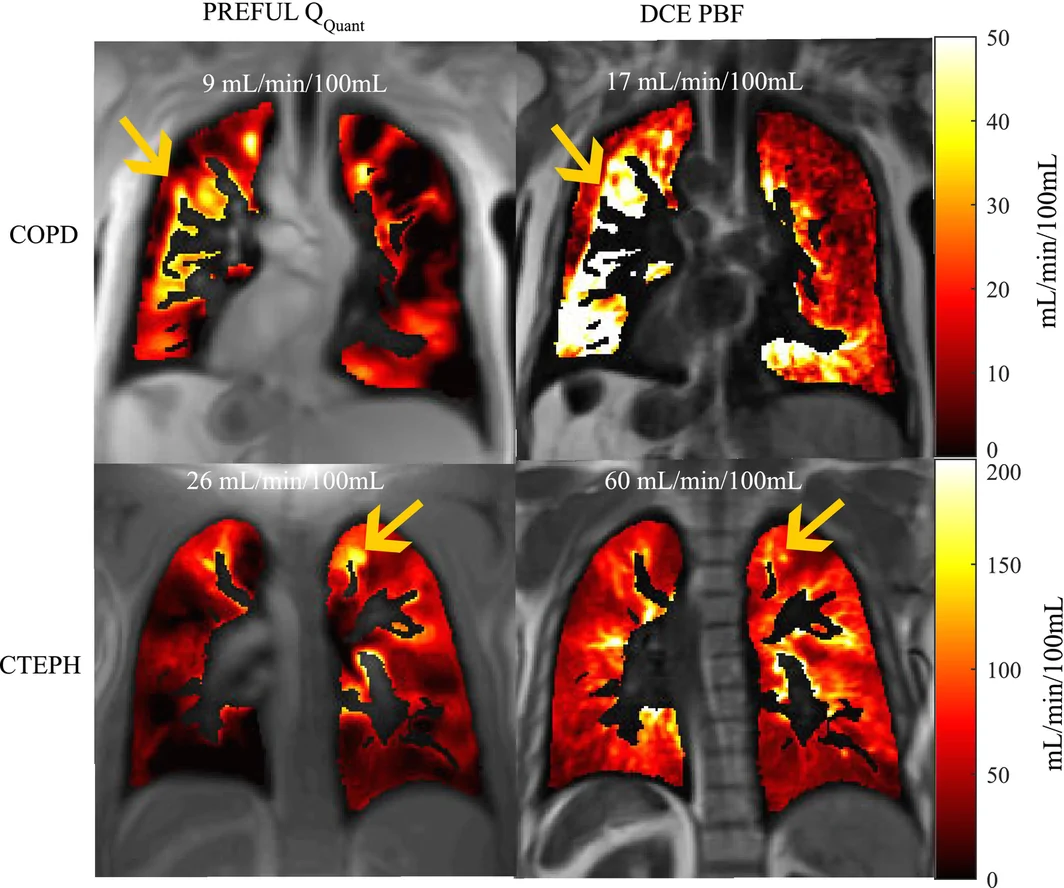
Perfusion maps derived by PREFUL MRI (Gaussian filtered ΔS, *σ* = 1.5) and DCE‐MRI of a representative 71‐year‐old male with COPD (top row) and a 56‐year‐old male with CTEPH (bottom row). While Q_Quant_ values are systematically lower than DCE PBF, localization of defect regions is similar, with better agreement in COPD than CTEPH. Nevertheless, PREFUL seems to overestimate defects compared to DCE. High perfused areas visible in both modalities are indicated with arrows.

Bland–Altman analysis (ΔS_GF1.5_ shown as an example in Figure 4, all plots are provided in Figure S3) shows consistent mean differences and limits of agreement across all ΔS options. In the COPD cohort, the median Q_Quant_ values ranged from 24 mL/min/100 mL (ΔS_regional_) to 19 mL/min/100 mL (ΔS_median_), with a median PBF value of 41 mL/min/100 mL. The CTEPH cohort showed in median lower Q_Quant_ values (14–16 mL/min/100 mL) while showing similar DCE PBF values (median 46 mL/min/100 mL).

**FIGURE 4:**
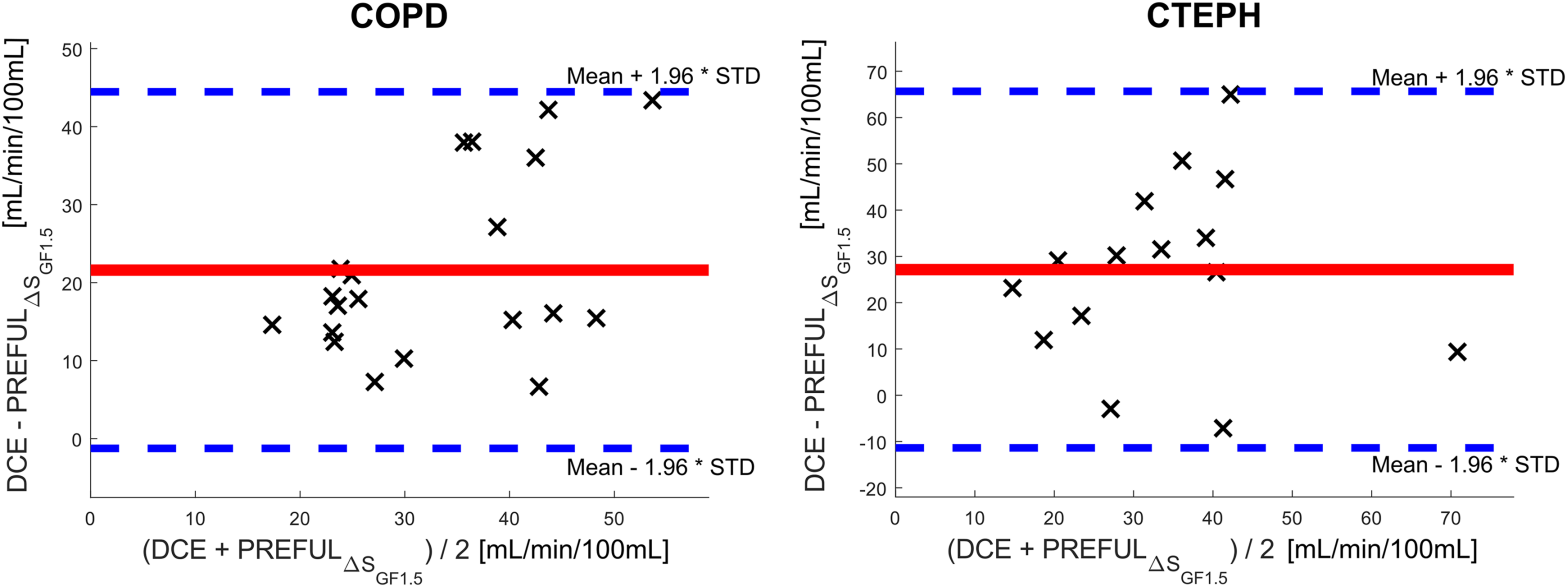
Bland–Altman plots of median Q_Quant_ and DCE PBF for all patients calculated with a Gaussian filtered (*σ* = 1.5) ΔS. COPD (left) shows lower mean bias and smaller limits of agreement than CTEPH (right).

Global Pearson correlation demonstrated similar moderate to strong positive association in COPD patients across all methods (all *r* > 0.5, all *p* ≤ 0.02). The strongest correlation was observed for ΔS_median_ with *r* = 0.59, followed by ΔS_GF1.5_ with *r* = 0.57. In the CTEPH cohort, the global correlation of Q_Quant_ was generally weaker (*r* > 0.28, *p* < 0.31), with the strongest correlations for regional and all Gauss filtered ΔS (*r* = 0.32). Voxel‐wise correlations showed greater variability between the ΔS methods. Highest voxel‐wise correlations were found with ΔS_GF1.5_ (COPD *r* > 0.21, CTEPH *r* > 0.35, both *p* ≤ 0.21) and ΔS_median_ (COPD *r* > 0.19, CTEPH *r* > 0.33, both *p* ≤ 0.25). All global and voxel‐wise Pearson correlations are presented in Tables 4 and 5.

**TABLE 4:** DCE PBF and PREFUL median (IQR) Q_Quant_ (mL/min/100 mL) using various ΔS filter options.

	DCE PBF	PREFUL ΔS_regional_ voxel‐wise regional	PREFUL ΔS_GF0.5_ GF *σ* = 0.5	PREFUL ΔS_GF1_ GF *σ* = 1	PREFUL ΔS_GF1.5_ GF *σ* = 1.5	PREFUL ΔS_median_ slice median
COPD cohort						
Global lung perfusion [mL/min/100 mL] (IQR)	41 (23)	24 (9)	22 (10)	21 (11)	20 (11)	19 (13)
Global Pearson correlation *r* (*p* value)	—	0.51 (0.02)	0.55 (0.01)	0.56 (0.01)	0.57 (0.01)	0.59 (0.01)
CTEPH cohort						
Global lung perfusion [mL/min/100 mL] (IQR)	46 (28)	16 (15)	16 (15)	15 (14)	15 (14)	14 (14)
Global Pearson correlation *r* (*p* value)	—	0.32 (0.25)	0.32 (0.25)	0.32 (0.25)	0.32 (0.25)	0.28 (0.31)

Abbreviations: COPD, chronic obstructive pulmonary disease; CTEPH, chronic thromboembolic pulmonary hypertension; DCE, dynamic contrast‐enhanced MRI; GF, Gaussian filter; PBF, pulmonary blood flow; PREFUL, phase‐resolved functional lung; ΔS, maximal blood exchange fraction.

**TABLE 5:** Median over the COPD and CTEPH cohort patients of DCE PBF and PREFUL voxel‐wise Pearson correlation coefficients (IQR), obtained using various PREFUL ΔS filter options for individual slice locations (dorsal, central, and ventral) and for all slices combined (whole lung).

ΔS option	Anterior	Central	Posterior	Whole lung
COPD cohort				
Voxel‐wise regional ΔS_regional_	−0.02 (0.15)	−0.04 (0.10)	−0.01 (0.13)	−0.02 (0.10)
GF *σ* = 0.5 ΔS_GF0.5_	0.02 (0.15)	0.03 (0.16)	0.03 (0.15)	0.00 (0.05)
GF *σ* = 1 ΔS_GF1_	0.12 (0.22)	0.14 (0.17)	0.15 (0.20)	0.10 (0.17)
GF *σ* = 1.5 ΔS_GF1.5_	0.22 (0.09)	0.34 (0.12)	0.36 (0.16)	0.32 (0.12)
Slice median ΔS_median_	0.20 (0.15)	0.35 (0.19)	0.37 (0.22)	0.32 (0.14)
CTEPH cohort				
Voxel‐wise regional ΔS_regional_	0.28 (0.39)	0.28 (0.38)	0.05 (0.42)	0.26 (0.41)
GF *σ* = 0.5 ΔS_GF0.5_	0.31 (0.35)	0.34 (0.26)	0.37 (0.46)	0.37 (0.23)
GF *σ* = 1 ΔS_GF1_	0.31 (0.36)	0.34 (0.29)	0.38 (0.45)	0.38 (0.23)
GF *σ* = 1.5 ΔS_GF1.5_	0.36 (0.35)	0.42 (0.22)	0.40 (0.43)	0.40 (0.21)
Slice median ΔS_median_	0.35 (0.36)	0.40 (0.23)	0.34 (0.40)	0.41 (0.25)

*Note:* All *p*‐values are < 0.001.

Abbreviations: COPD, chronic obstructive pulmonary disease; CTEPH, chronic thromboembolic pulmonary hypertension; GF, Gaussian filter; ΔS, maximal blood exchange fraction.

### 3.3 Perfusion Gradient in Anterior–Posterior Direction

Given the previous results and comparable performance across ΔS filtering methods, the subsequent analysis of perfusion gradient in anterior–posterior direction is presented only for ΔS_GF1.5_. Results for the other approaches are provided in Table S1. All voxel Q_Quant_ values (per slice) of the overall dataset (representative also for individual subjects) are shown in Figure 5 (left). The most gravity‐dependent (posterior) slice consistently demonstrated the highest perfusion values, with both the highest individual Q_Quant_ values and the highest median values, with an overall median of 41 mL/min/100 mL, being significantly higher than the most anterior slice.

**FIGURE 5:**
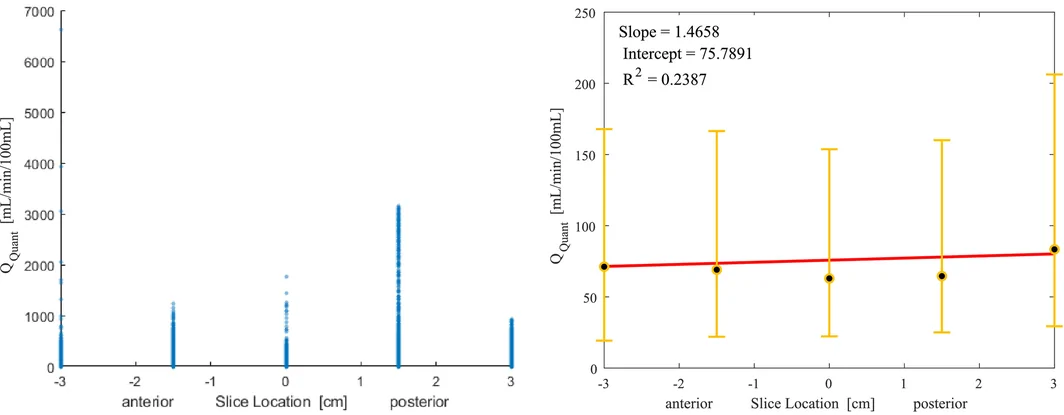
Q_Quant_ values using Gaussian filter with *σ* = 1.5 by slice location. Left: healthy subjects Q_Quant_ per voxel; right: median (IQR) values per slice including linear fit. General highest perfusion values were observed in the second posterior slice, highest median in the posterior slice. High outliers in some voxels likely originate from remaining vessel parts.

The distribution of overall slice‐wise median Q_Quant_ values and the corresponding median of linear fits per patient is presented in Figure 5 (right), resulting in an overall slope of 1.47 mL/min/100 mL/cm and a strength of the linear relationship with *R* ^2^ = 0.24, relative to the posterior slice, resulting in a change of 1.8%/cm.

## 4 Discussion

This study introduces an optimized PREFUL perfusion quantification method that filters ΔS values to preserve greater spatial information during the calculation. We validated the findings by improving intrascan and interscan repeatability in healthy subjects while maintaining global and voxel‐wise Q_Quant_ correlation to DCE PBF in similar range in COPD and CTEPH patients. However, PREFUL Q_Quant_ systematically underestimated absolute perfusion relative to DCE and also yielded lower values than other quantification methods in healthy subjects. Furthermore, we showed that with PREFUL Q_Quant_ the expected gravity‐dependent perfusion gradient can be observed in healthy volunteers, indicating reliability across slices and supporting physiologically plausible PREFUL perfusion.

### 4.1 Intrascan and Interscan Repeatability of Q_Quant_ Under Varying ΔS Conditions in Healthy Volunteers

Applying different filters to ΔS improved the repeatability of Q_Quant_, with CoV values comparable to those reported in a previous study [20]. However, filtering settings below *σ* = 1.5 still produced image artifacts, even in healthy volunteers with expected homogeneous Q_Quant_, hindering reliable quantification of perfusion. Differences between intrascan (identical slice positions) and interscan results underscore sensitivity of Q_Quant_ to minor variations in positioning or vessel segmentation. We also acknowledge that the heart frequency was omitted from the Q_Quant_ calculation for repeatability analysis because of its considerable variability across scans. This effect might be due to habituation effects, with healthy volunteers becoming more relaxed over the course of the repeatability scans [21].

### 4.2 Comparison of PREFUL and DCE‐MRI

The results of this study also demonstrate a moderate to strong positive correlation between the Q_Quant_ and DCE‐derived perfusion values across all filtering options, which are in agreement with previous DCE‐MRI correlations [8, 15]. Q_Quant_ values were consistently lower in the CTEPH cohort than in the COPD cohort, whereas DCE PBF showed the inverse pattern. These differences likely arise because the technique captures disease‐specific characteristics with varying effectiveness, as previously reported [8, 15]. In CTEPH, web‐like stenoses in the pulmonary arteries reduce blood flow to distal vessels leading to a reduced blood flow velocity. The result is a phase shift between obstructed and nonobstructed lung segments. As PREFUL Q_Quant_ relies only on one phase with the maximum flow‐related signal in the whole lung, this will likely result in an underestimation of perfusion in obstructed regions and in all lung areas exhibiting a phase delay relative to the selected phase. While previous studies have shown a moderate to strong correlation for perfusion defect percentage between DCE and PREFUL also in CTEPH patients [15, 22, 23], the overall underestimation of Q_Quant_ might lead to an increased divergence to DCE measurements, generally making PREFUL Q_Quant_ as a standalone value less reliable in patients with CTEPH than in those with other conditions, such as COPD.

Q_Quant_ values were generally lower than DCE‐derived PBF values. This difference, also reflected in the median values and in the mean bias, suggests a systematic underestimation of Q_Quant_ derived by PREFUL MRI. These findings align with other lung perfusion quantification studies, which consistently report higher average values than our results across both healthy cohorts and diseased patients. For instance, an alternative PREFUL MRI quantification approach yielded perfusion values > 86 mL/min/100 mL in healthy volunteers and in COPD patients [20], and ASL studies reported values exceeding 100 mL/min/100 mL in healthy and diseased lungs [24, 25]. Possible reasons for this underestimation include that PREFUL is prone to show more perfusion defects due to low signal‐to‐noise ratio and the intrinsic sensitivity only to flow that is orthogonal to the image slice, so perfusion flow predominantly in‐plane may be missed. Other errors may arise from suboptimal fitting of the signal decay during the ΔS calculation or from image registration inaccuracies, particularly in slices that contain a large portion of the heart.

### 4.3 Perfusion Gradient in Anterior–Posterior Direction

The observed slope of 1.8%/cm is within the range of previously reported lung perfusion changes values such as 3%/cm^11^ in sagittal slice, a slope of 0.447 between middle and posterior slice (meaning about 0.15%/cm) [18], or lower than observed 14%/cm in sagittal slices [19]. The differences might be due to the following reasons.

One might be due to incomplete coverage of the whole lung. In previous literature [10, 18, 26], mostly sagittal images spanning the full lung are used to assess gravitational gradients, making local effects more prone in our study. Differences in spatial resolution and blurring, together with greater vessel visibility in coronal compared to sagittal orientations, may result in more vessels being excluded in PREFUL images, potentially underestimating perfusion in some regions.

Additionally, inspiration status has been shown to influence the magnitude of the gravity‐dependent perfusion gradient, with lower values observed in expiration [18]. This effect cannot be accounted for in our study as all images were acquired in free breathing and registered towards a respective middle respiratory image.

The Q_Quant_ distribution over slice position also matches prior findings, where a strong linear correlation only occurred when calculating perfusion/density and the highest perfusion was observed a more intermediate than posterior part [10]. However, the middle slice exhibited fewer voxels with high Q_Quant_ values than expected, likely due to omission of large vessels. Although no additional vessel segmentation was performed, prominent vascular structures near the parenchymal borders were still inadvertently excluded from the analysis by the automated processing.

## 5 Limitations

This study has several limitations. First, the number of acquired slices (3–5) for PREFUL MRI was relatively low, especially for assessing the gravity‐dependent perfusion gradient. While the acquired PREFUL slices are representative of the lung, future studies should consider acquiring additional coronal and preferably sagittal slices to capture the entire lung volume when assessing gravity‐induced perfusion gradients. This would enable a more comprehensive evaluation of the gradient and allow direct comparison with studies that assess gradients in the sagittal plane. Besides, sagittal slices would allow investigation of the underestimation of perfusion that may arise from predominantly in‐plane blood flow. Additionally, incorporating lung density corrections [26] could improve the accuracy of Q_Quant_‐based gradient measurements.

The comparison between Q_Quant_ and DCE‐MRI is inherently limited by differences in spatial and temporal resolution, as PREFUL only acquired three slices, whereas DCE as a 3D sequence covers the whole lung, which can only be partially accounted for by using a compound DCE slice. DCE‐derived PBF values may also vary depending on the quantification method [27, 28, 29] and breathing position [30]. While the methods seem similar to a previous study, where almost no bias was detected [8], slight changes in the quantification algorithms might introduce a greater bias. Further errors might occur due to the registration of PREFUL images to DCE. Inaccurate registration might lead to artifacts, particularly problematic in the lower lung, where PREFUL images are acquired during tidal breathing, whereas DCE is performed during breath hold at end inspiration. Furthermore, DCE itself may be prone to errors in this region, as patients often struggle to maintain breath holds during acquisition. Further validation against gold‐standard methods, such as SPECT, would be beneficial, mirroring the approach taken in previous Q_Quant_ evaluations [15].

The selected patient groups limit the scope of the evaluation. The healthy volunteers were considerably younger than the diseased cohorts, and although CTEPH already exhibits distinct perfusion patterns compared with healthy and COPD lungs, the study did not include other clinically relevant pulmonary conditions. Although we expect the observed underestimation of perfusion to be specific to CTEPH, inclusion of a broader range of disease cohorts would be beneficial in future work.

While filtering ΔS improved regional information conservation without compromising correlation to DCE‐MRI, other parameters in the Q_Quant_ calculation (e.g., heart rate) remain potential sources of variability.

Beyond reducing ΔS‐related artifacts, further improvements could be achieved by refining the signal decay fitting and adopting a different phase‐selection strategy. For example, incorporating a broader range of cardiac phases, rather than the current single‐phase approach, might better capture perfusion in CTEPH patients, whose perfusion patterns are not accurately represented with the existing method.

## 6 Conclusion

Applying a Gaussian filter with *σ* = 1.5 to ΔS in Q_Quant_ calculation improves repeatability and maintains correlation to DCE. Consequently, this filtering approach is recommended to achieve more stable and robust quantification in PREFUL‐derived perfusion analysis. Furthermore, the gravity‐dependent perfusion gradient can be observed using PREFUL MRI in healthy volunteers, suggesting interslice consistency and physiological plausible results. While these findings represent a step towards reliable, quantified perfusion measurements, further improvements in CTEPH assessment and testing in larger, more diverse cohorts, preferably with extended lung coverage, are necessary to confirm its robustness across a broader range of clinical scenarios.

## Author Contributions

**Kirsten J. Schmidt:** data curation, investigation, formal analysis, software, visualization, methodology, writing – original draft. **Andreas Voskrebenzev:** conceptualization, software, methodology, supervision, writing – review and editing. **Julian M. W. Glandorf:** methodology, writing – review and editing. **Marius M. Klein:** data curation, investigation, writing – review and editing. **Lea Behrendt:** data curation, methodology, software, writing – review and editing. **Frank Wacker:** funding acquisition, resources, writing – review and editing. **Jens Vogel‐Claussen:** conceptualization, funding acquisition, resources, supervision, writing – review and editing. **Filip Klimeš:** conceptualization, data curation, methodology, supervision, writing – review and editing.

## Funding

This work was funded by the German Center for Lung Research (DZL).

## Conflicts of Interest

F.K., A.V., and J.V.‐C. are shareholders of BioVisioneers GmbH, a company that has an interest in pulmonary magnetic resonance imaging methods. M.M.K. is an employee of BioVisioneers GmbH, but did not receive funding for this research. The remaining authors have no conflicts of interest to declare.

## Supporting information


**Figure S1:** Exemplary ΔS and QQuant maps from a healthy male volunteer (26 years) demonstrating the effects of different filter settings applied during ΔS calculation. Without filtering small artifacts are visible.
**Figure S2:** Bland–Altman plots of heart–rate–normalized perfusion measurements for each ΔS options. Reported mean differences with Limits of Agreement (LoA) are as follows: ΔS voxel‐wise regional, intrascan mean difference 5.6 mL/100 mL (LoA: [−24.6, 35.9]) and interscan mean difference 3.3 mL/100 mL (LoA: [−23.5, 30.2]), ΔS GF *σ* = 0.5 mean difference 3.3 mL/100 mL (LoA: [−24.8, 31.4]) and interscan mean difference 3.0 mL/100 mL (LoA: [−24.0, 30.0]), ΔS GF *σ* = 1 intrascan mean difference 3.1 mL/100 mL (LoA: [−25.1, 31.3]) and interscan mean bias 3.0 mL/100 mL (CI:[−23.5, 29.5]), ΔS GF *σ* = 1.5 intrascan mean difference 2.9 mL/100 mL (LoA: [25.0, 30.9]) and interscan mean difference 2.9 mL/100 mL (LoA: [−23.1, 29.0]), and ΔS as a slice median intrascan mean difference 5.3 mL/min/100 mL (LoA: [−23.1, 33.7]) interscan mean difference 3.8 mL/100 mL (LoA: [−20.7, 28.2]).
**Figure S3:** Bland–Altman plots of median Q_Quant_ and DCE PBF for all ΔS options.
**Table S1:** Median slice Q_Quant_ [mL/min/100 mL] (IQR) from anterior slice 1 to posterior slice 5 of the healthy repeatability cohort, as well as the median slope of the linear regression for each subject for all ΔS options.
**Table S2:** Time between heartbeats T [s] for each healthy volunteer in the repeatability scans. On median, the subjects time between heartbeat increases with following scans, suggesting habituation. The increase was significantly higher in B1 and B2 than in A1, increase to A2 values was not significant (*p* = 0.1).
**Table S3:** Median Q_Quant_, in intrascan (A) and interscan (B) repeatability scans.

## Data Availability

The data that support the findings of this study are available from the corresponding author upon reasonable request.
