# Supplementary material for: Evaluating Perfusion Quantification for Phase‐Resolved Functional Lung MRI Using Gaussian Filtering
Source: NMR Biomed. 2026 Oct 13;39(11):e70420. doi: 10.1002/nbm.70420 (PMC13659365; doi:10.1002/nbm.70420)
Supplement: Supplementary file 1 [file NBM-39-e70420-s001.docx]

# Supporting Information

Figure S1 Exemplary ΔS and QQuant maps from a healthy male volunteer (26 years) demonstrating the effects of different filter settings applied during ΔS calculation. Without filtering small artifacts are visible.

Figure S2 Bland‑Altman plots of heart‑rate‑normalized perfusion measurements for each ΔS options. Reported mean differences with limits of agreement (LoA) are as follows: ΔS voxel-wise regional, intrascan mean difference 5.6mL/100mL (LoA: [-24.6,35.9]) and interscan mean difference 3.3mL/100mL (LoA: [-23.5,30.2]), ΔS GF σ=0.5 mean difference 3.3mL/100mL (LoA: [-24.8,31.4]) and interscan mean difference 3.0mL/100mL (LoA: [-24.0,30.0]), ΔS GF σ=1 intrascan mean difference 3.1mL/100mL(LoA: [-25.1,31.3]) and interscan mean bias 3.0mL/100mL (LoA:[-23.5,29.5]), ΔS GF σ=1.5 intrascan mean difference 2.9mL/100mL (LoA: [25.0,30.9] ) and interscan mean difference 2.9mL/100mL (LoA: [-23.1,29.0]) and ΔS as a slice median intrascan mean difference 5.3mL/min/100mL (LoA: [-23.1,33.7]) interscan mean difference 3.8mL/100mL (LoA: [-20.7,28.2]).

Figure S3 Bland-Altman plots of median Q_Quant_ and DCE PBF for all ΔS options.

*Table S1 Median slice QQuant [mL/min/100mL] (IQR) from anterior slice 1 to posterior slice 5 of the healthy repeatability cohort, as well as the median slope of the linear regression for each subject for all ΔS options.*

|  | **QQuant [mL/min/100mL]** | | | | | |
| --- | --- | --- | --- | --- | --- | --- |
| **ΔS variant** | Slice 1 anterior | Slice 2 | Slice 3 | Slice 4 | Slice 5 posterior | Median Slope |
| Voxel-wise regional ΔS_regional_ | 73 (28) | 71 (27) | 64 (38) | 67 (35) | 85 (55) | 1.44 |
| GF σ=0.5 ΔS_GF0.5_ | 73 (29) | 70 (26) | 63 (38) | 66 (35) | 84 (55) | 1.44 |
| GF σ=1 ΔS_GF1_ | 72 (29) | 69 (26) | 63 (38) | 65 (35) | 84 (54) | 1.45 |
| GF σ=1.5 ΔS_GF1.5_ | 70 (29) | 68 (25) | 62 (37) | 65 (36) | 83 (54) | 1.47 |
| Slice median ΔS_median_ | 67 (29) | 65 (26) | 58 (38) | 60 (35) | 78 (54) | 1.26 |

Table S2 Time between heartbeats T [s] for each healthy volunteer in the repeatability scans. On median the subjects time between heartbeat increases with following scans, suggesting habituation. The increase was significantly higher in B1 and B2 than in A1, increase to A2 values was not significant (p=0.1).

| Subject | A1  T [s] | A2  T [s] | B1  T [s] | B2  T [s] |
| --- | --- | --- | --- | --- |
| P1 | 0.95 | 0.89 | 0.95 | 0.89 |
| P2 | 0.96 | 0.96 | 1.00 | 1.05 |
| P3 | 1.00 | 1.09 | 0.96 | 0.98 |
| P4 | 0.83 | 0.81 | 0.85 | 0.77 |
| P5 | 0.81 | 0.82 | 0.88 | 0.88 |
| P6 | 0.89 | 0.83 | 0.88 | 0.91 |
| P7 | 0.89 | 0.93 | 0.96 | 1.02 |
| P8 | 1.05 | 1.20 | 1.12 | 1.09 |
| P9 | 0.81 | 0.86 | 0.93 | 0.83 |
| P10 | 1.23 | 1.05 | 1.17 | 1.20 |
| P11 | 0.71 | 0.76 | 0.81 | 0.76 |
| P12 | 0.96 | 0.98 | 1.14 | 1.20 |
| P13 | 0.74 | 1.00 | 0.83 | 0.96 |
| P14 | 0.69 | 0.67 | 0.74 | 0.76 |
| P15 | 0.78 | 0.40 | 0.82 | 0.85 |
| P16 | 0.70 | 0.72 | 0.81 | 0.72 |
| P17 | 0.82 | 0.91 | 0.88 | 0.88 |
| P18 | 0.76 | 0.89 | 0.91 | 1.00 |
| P19 | 0.83 | 0.95 | 1.26 | 1.07 |
| P20 | 1.02 | 0.98 | 1.00 | 1.14 |
| P21 | 1.17 | 1.29 | 1.12 | 1.33 |
| P22 | 0.89 | 1.00 | 1.07 | 0.93 |
| P23 | 0.93 | 0.98 | 1.07 | 1.09 |
| P24 | 1.23 | 1.20 | 1.26 | 1.37 |
| P25 | 0.83 | 0.91 | 0.81 | 0.88 |
| P26 | 0.85 | 0.86 | 0.96 | 1.00 |
| P27 | 0.88 | 0.95 | 0.96 | 1.02 |
| P28 | 0.81 | 0.81 | 0.89 | 0.89 |
| P29 | 0.95 | 1.09 | 1.07 | 1.26 |
| median | 0.88 | 0.93 | 0.96 | 0.98 |

Table S3 Median Q_Quant_, in intrascan (A) and interscan (B) repeatability scans.

| **A. Intrascan** |  |  |  |  |  |  |
| --- | --- | --- | --- | --- | --- | --- |
| **ΔS Option** | **Median QQuant**  **A1 [mL/min/100mL]**  **(IQR)** | **Median QQuant**  **A2 [mL/min/100mL] (IQR)** | **Median CoV [%] (IQR)** | **Mean bias [mL/min/100mL]** | **Wilcoxon p Value** | **Cohen’s d** |
| Voxel-wise  regional ΔS_regional_ | 81 (29) | 68 (19) | 8.66 (7.93) | 8.86 | 0.002 | 0.65 |
| GF σ=0.5 ΔS_GF0.5_ | 75 (24) | 69 (17) | 9.61 (10.41) | 4.97 | 0.01 | 0.52 |
| GF σ=1 ΔS_GF1_ | 75 (25) | 69 (17) | 8.52 (11.47) | 5.76 | 0.02 | 0.50 |
| GF σ=1.5 ΔS_GF1.5_ | 74 (25) | 68 (17) | 8.70 (11.47) | 5.52 | 0.03 | 0.49 |
| Slice median ΔS_median_ | 70 (28) | 62 (19) | 8.46 (8.99) | 8.21 | 0.004 | 0.62 |
| **B. Interscan** |  |  |  |  |  |  |
| **ΔS Option** | **Median QQuant**  **A1 [mL/min/100mL] (IQR)** | **Median QQuant**  **B1 [mL/min/100mL] (IQR)** | **Median CoV [%] (IQR)** | **Mean bias [mL/min/100mL]** | **Wilcoxon (p Value)** | **Cohen’s d** |
| Voxel-wise  regional ΔS_regional_ | 81 (29) | 67 (19) | 11.67 (12.38) | 9.44 | 0.002 | 0.61 |
| GF σ=0.5 ΔS_GF0.5_ | 75 (24) | 65 (17) | 9.96 (13.42) | 6.49 | 0.003 | 0.60 |
| GF σ=1 ΔS_GF1_ | 75 (25) | 65 (19) | 10.79 (13.43) | 8.90 | 0.003 | 0.60 |
| GF σ=1.5 ΔS_GF1.5_ | 74 (25) | 66 (20) | 10.60 (13.63) | 8.75 | 0.004 | 0.60 |
| Slice median ΔS_median_ | 70 (28) | 63 (18) | 11.53 (11.14) | 9.32 | 0.001 | 0.64 |
| CoV, coefficient of variation; ΔS, maximal blood exchange fraction; GF, gaussian filter; IQR, interquartile range; QQuant_norm_, quantified perfusion normalized by heart frequency.  Statistically significant values are marked with *. | | | | | | |
